# Unusual Developmental Vascular Anomalies: Insights From a Chest Physician’s Perspective

**DOI:** 10.7759/cureus.65802

**Published:** 2024-07-30

**Authors:** Shaz Assain, Subramanian S, Gokulakrishnan Sekar, Mohanabalamurugan V

**Affiliations:** 1 Department of Respiratory Medicine, SRM Medical College Hospital and Research Centre, SRM Institute of Science and Technology (SRMIST), Chennai, IND; 2 Department of Respiratory Medicine, Meenakshi Medical College Hospital and Research Institute, Kanchipuram, IND

**Keywords:** trail's sign, mitral stenosis, developmental vascular anomalies, congenital anomalies, pulmonary artery agenesis

## Abstract

This case report discusses three developmental vascular anomalies (DVAs) observed in adults and highlights the challenges related to the diagnosis and management. Even though detected at early ages, diagnostic difficulties are observed in the adult age due to the scarcity and diverse clinical features. These cases illustrate the necessity of a multidisciplinary approach involving clinicians and radiologists for precise and prompt diagnosis in adults, where misdiagnosis and delays in intervention are frequent. The cases comprised a 17-year-old female with an absent right pulmonary artery and mitral stenosis, a 46-year-old female with chronic obstructive pulmonary disease (COPD), with an absent left pulmonary artery, and a 60-year-old female with bronchial asthma and tuberculosis exhibiting a rare DVA. This discussion highlights the importance of intensified clinical suspicion and thorough evaluation for the cases of unexplained respiratory symptoms and abnormal image findings in patients, which can further provide the medical community with valuable insights.

## Introduction

Developmental vascular anomalies (DVAs) encompass a heterogeneous spectrum of conditions with diverse clinical and radiological manifestations and are typically diagnosed during prenatal and early childhood. Identifying DVAs in adults poses a diagnostic challenge because of their rarity and varied clinical presentation. In this study, three compelling cases shed light on the complexities associated with DVAs in mature individuals. DVAs arise from normal embryonic development disruptions, ranging from being asymptomatic and incidentally discovered during imaging studies to presenting with symptoms like cough, hemoptysis, and recurrent pneumonia. Particularly in the rare cases of late presentation of DVAs, the absence or lack of distinguishing characteristics and typical clinical presentation can often result in misdiagnosis and delay the appropriate and necessary interventions [1-3].

Recognizing the unique radiological features of DVAs is crucial in managing mature patients, as they serve as primary clues for determining clinical diagnoses [2,3]. The three cases discussed in this report highlight DVAs observed in adult patients. Case 1 discusses the absent right pulmonary artery and the presence of mitral stenosis in a 17-year-old female experiencing exertional dyspnea. Case 2 describes a 46-year-old female with an absent left pulmonary artery and chronic obstructive pulmonary disease (COPD). Case 3 discusses a 60-year-old female with bronchial asthma, pulmonary tuberculosis, persistent left superior vena cava, and an aberrant right subclavian artery (also known as arteria lusoria) [4], who presented with loss of lung volume suspicious of pulmonary hypoplasia.

These cases focus on the clinical diversity of DVAs and highlight the critical need for a multidisciplinary strategy involving clinicians and radiologists. Since the literature on delayed diagnosis of DVAs is limited, this type of approach is crucial for achieving accurate and timely diagnosis in patients. This case report aims to contribute valuable insights into the medical community, raise awareness, and urge a high index of suspicion when an adult patient's unexplained respiratory symptoms coincide with abnormal imaging findings. Through a thorough exploration of the different cases, this study also aims to enhance the knowledge and understanding of these rare anomalies and early recognition for better patient outcomes.

## Case presentation

Case 1

**Figure 1:**
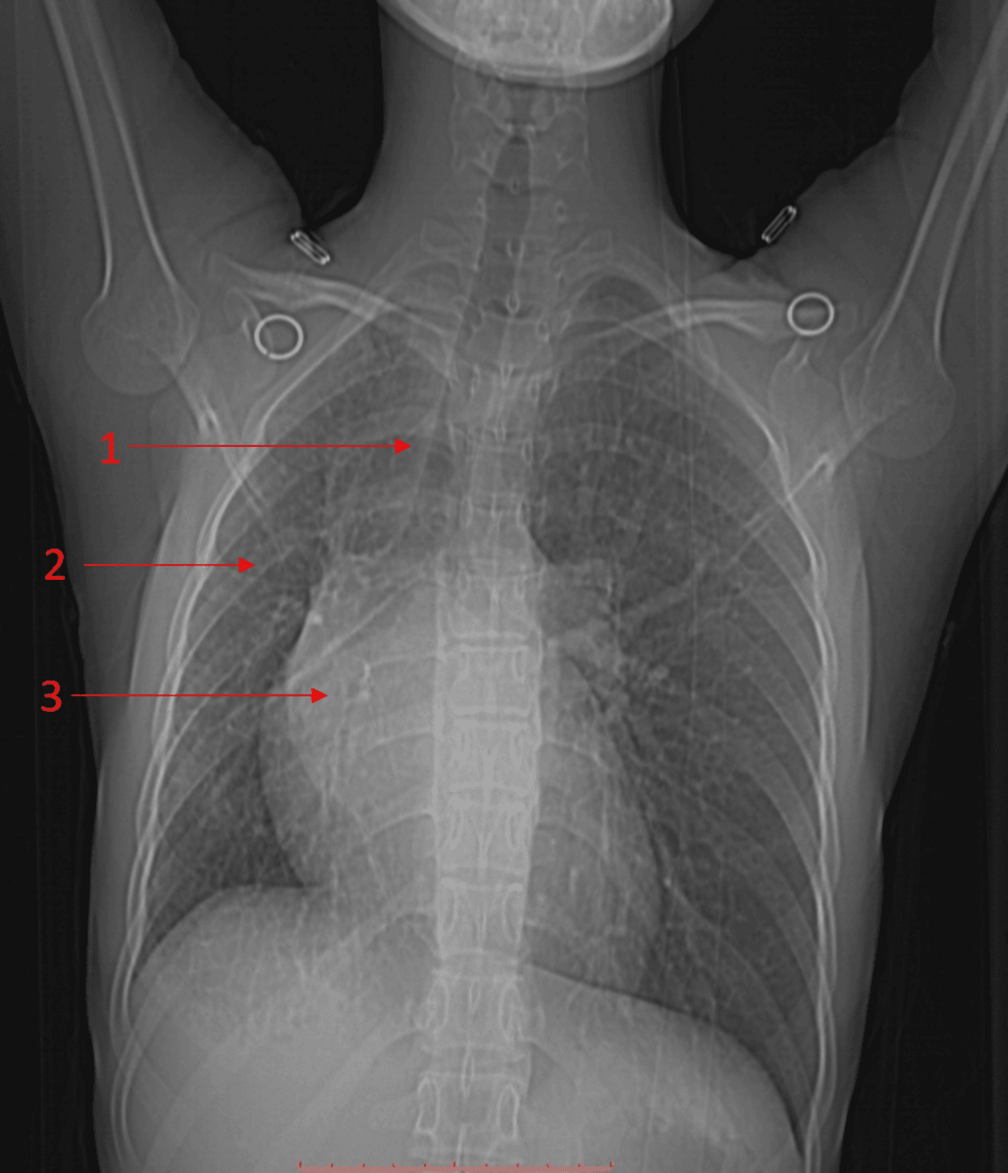
Case 1: X-ray and scout film 1 indicates the tracheal and mediastinal deviation to the right side. 2 indicates the volume loss of the right lung. 3 indicates the obscured right hilar shadow.

A 17-year-old female presented with exertional dyspnea lasting two years, rated as grade II on the Modified Medical Research Council (m-MRC) scale. This scale provides a standardized way to assess the severity of dyspnea and allows healthcare providers to quantify and monitor the changes in dyspnea over time. Despite the absence of typical respiratory symptoms, the clinical evaluation revealed reduced right chest expansion, positive Trail's sign (undue prominence of the clavicular head of sternomastoid on the side to which the trachea is shifted), and cardiovascular findings, including a loud S1 and mid-diastolic murmur in the mitral area. Chest X-ray (Figure 1) revealed tracheal deviation, obscured hilar shadows, and loss of lung volume on the right side.

**Figure 2:**
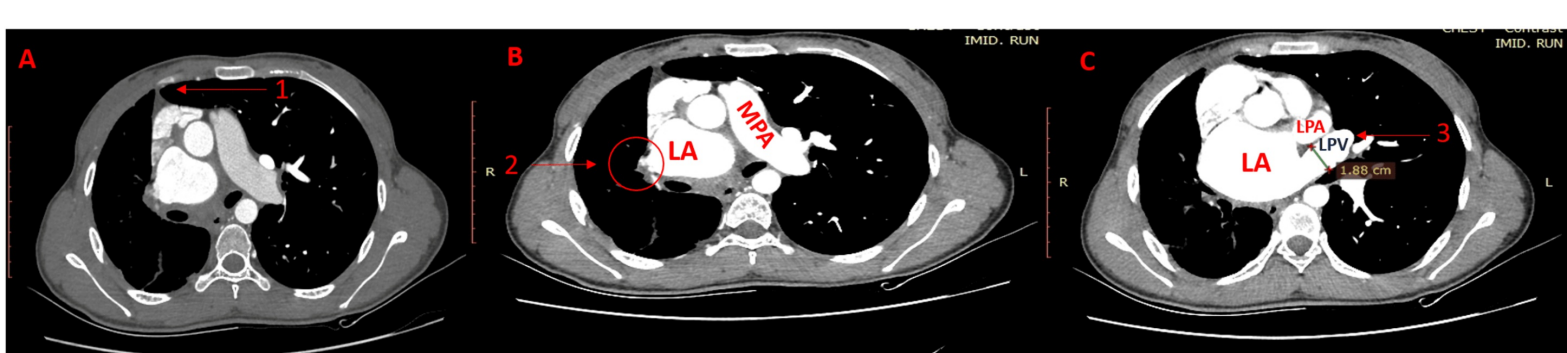
Contrast-enhanced CT scan (A) Herniation of the left lung (1). (B) Reduced caliber of the right pulmonary vein (2), dilated left atrium (LA), and main pulmonary artery (MPA). (C) Dilated left pulmonary vein (LPV) (3), dilated left atrium (LA), and left pulmonary artery (LPA).

Contrast-enhanced CT (Figure 2, Panels A-C) confirmed the absent right pulmonary artery. In adult patients, hemoptysis is the most common manifestation, and the risks of pulmonary hypertension (PHT), hemoptysis, and mortality significantly increase with age [5]. In this case, the valuable insights into DVAs are highlighted by providing important insights into the necessity of thorough imaging and clinical correlation for accurate diagnosis. It also provides insight into the importance of early detection of anomalies in individuals with unexplained respiratory symptoms because it is crucial for optimal intervention and patient outcome.

Case 2

A 46-year-old female with COPD presented with worsening dyspnea grade III on the m-MRC scale and cough with expectoration not associated with hemoptysis. Clinical examination revealed a positive Trail's sign, reduced chest expansion, and bilateral wheezing heard on auscultation over bilateral lung fields.

**Figure 3:**
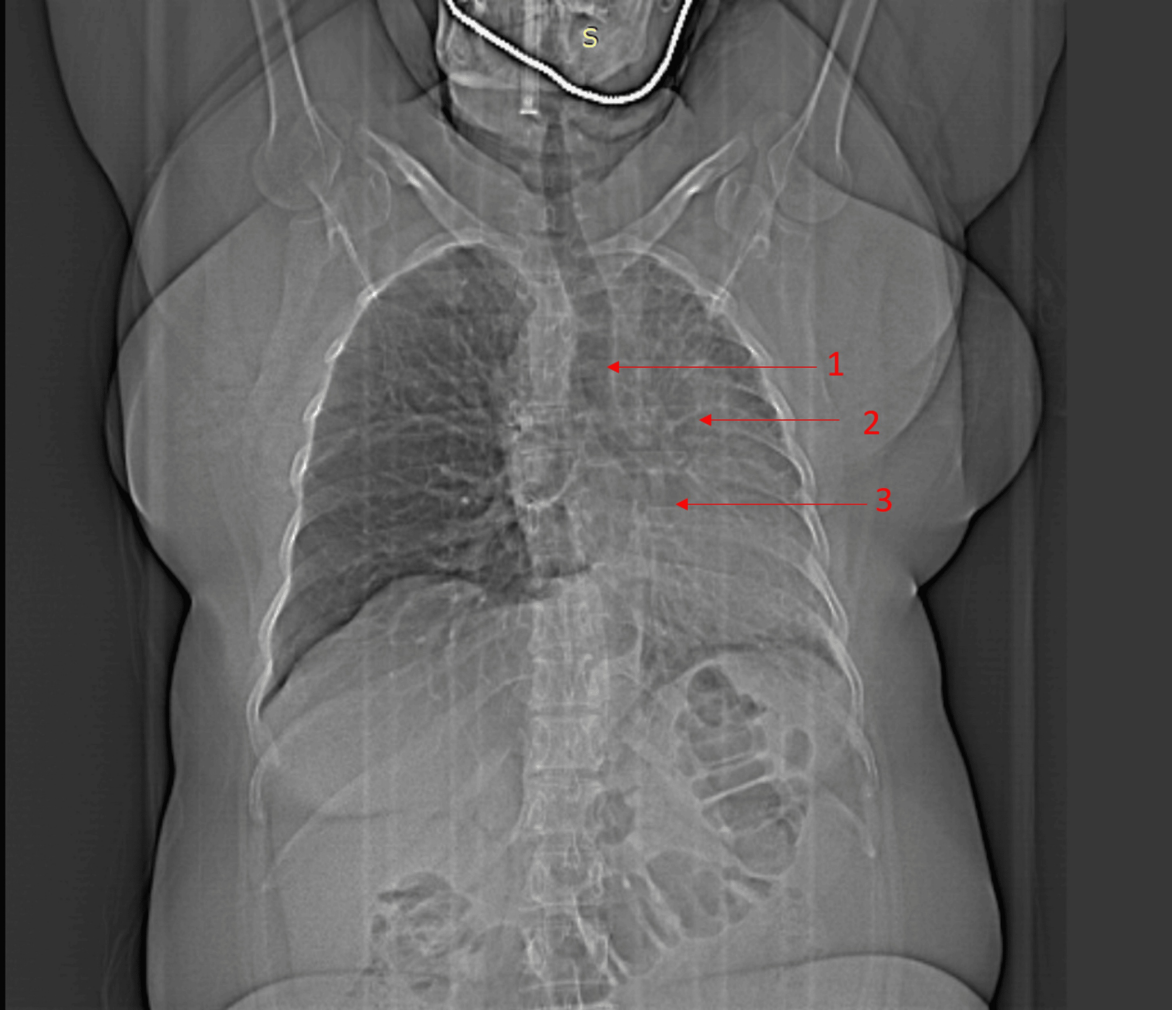
Case 2: X-ray and scout film 1 shows the trachea and mediastinal deviation to the left side. 2 shows the reduced lung volume on the left side. 3 shows the obscured hilar shadow on the left side.

The diagnosis included COPD coexisting with a rare anomaly, the absent left pulmonary artery (LPA). With a prevalence of 1/200,000, isolated unilateral absent pulmonary artery (UAPA) exhibits risks like recurrent infections and PHT in childhood. Imaging studies (Figure 3) revealed tracheal deviation, obscured hilar shadows, and loss of lung volume on the left side.

**Figure 4:**
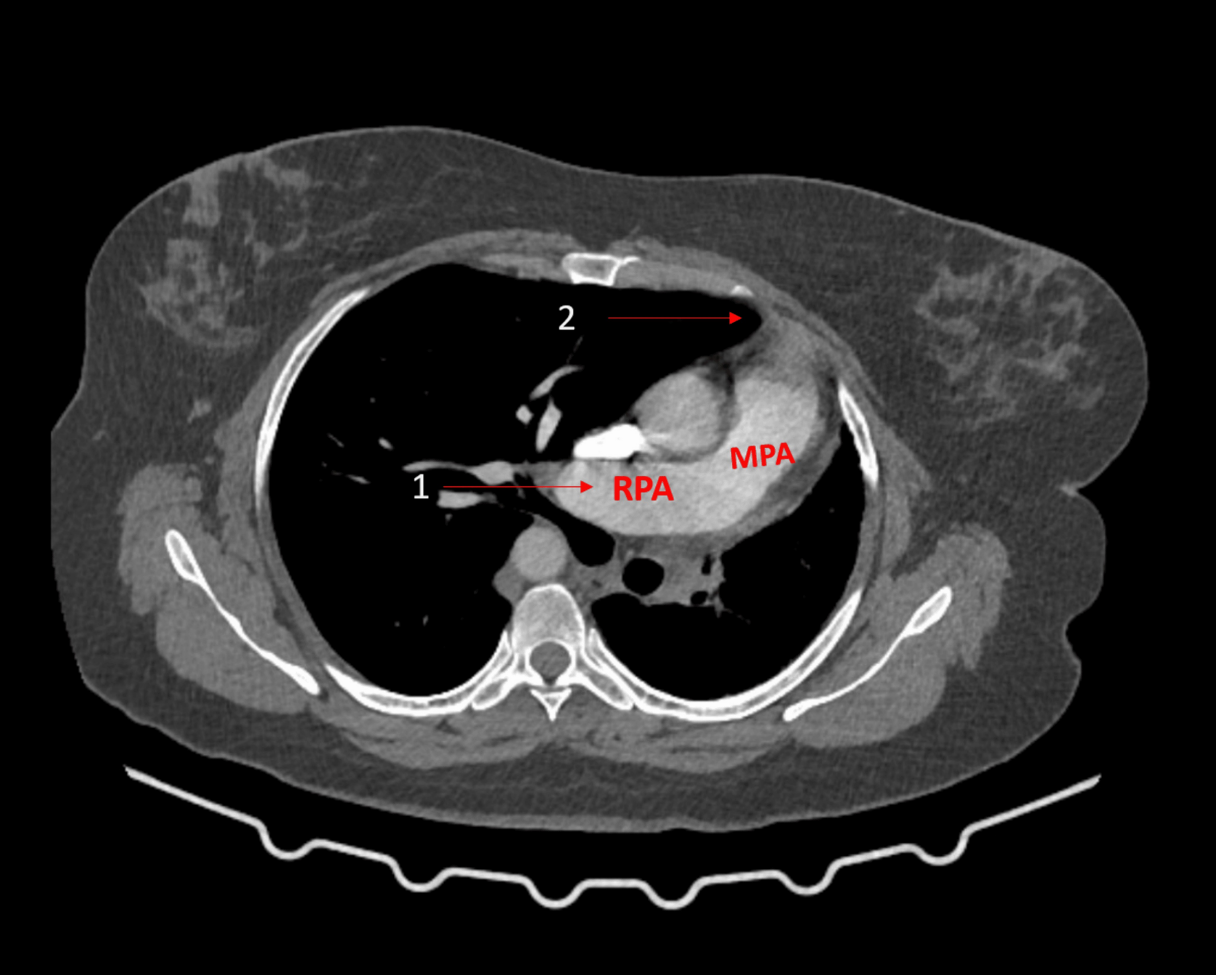
CT pulmonary angiogram 1 shows the right pulmonary artery (RPA) and main pulmonary artery (MPA). 2 shows the herniation of the right lung.

CT pulmonary angiogram (Figure 4) shows the absence of the LPA and herniation of the right lung. Patients are usually followed up regularly, and surgical interventions are required when a patient exhibits severe symptoms. Complications such as respiratory failure and massive pulmonary hemorrhage can occur with a mortality rate of 7%. This case report focuses on the necessity of a comprehensive approach for the recognition and management of patients with complex DVAs.

Case 3

A 60-year-old female with long-term bronchial asthma initiated anti-tubercular therapy for smear-positive pulmonary tuberculosis presented with dyspnea grade III on the m-MRC scale and productive cough. On clinical examination, clubbing and pallor were noticed. On auscultation, bilateral crackles were heard over bilateral lung fields.

**Figure 5:**
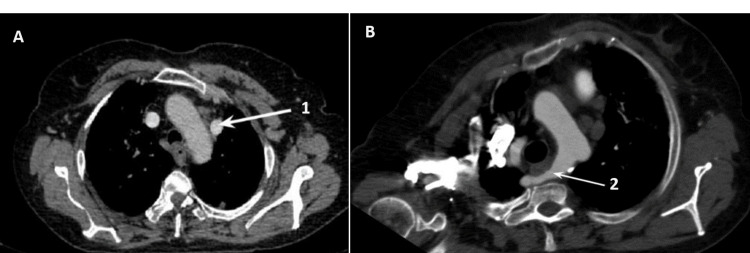
CT scan (A) Left superior vena cava (1). (B) Aberrant right subclavian artery (2).

Imaging findings (Figure 5, Panels A, B) revealed a left superior vena cava (SVC) and an aberrant right subclavian artery (RSA), a rare incidental finding. Asymptomatic cases of aberrant subclavian artery require follow-up. Surgical intervention is indicated for all patients who have symptoms such as dysphagia lusoria (esophageal compression caused by an aberrant RSA), cough, stridor, or aneurysmal aberrant RSA. Initially, treatment for aberrant RSA consisted of ligation of the vessel. Reports of ischemia and subclavian steal syndrome in open surgical correction of dysphagia lusoria were noted; therefore, the re-establishment of flow by connecting the divided subclavian artery either to the ascending aorta or to the right common carotid artery using a short interposition graft is recommended. Endovascular occlusion of aberrant RSA appears to be valuable in treating elderly patients with comorbidities that make them unsuitable for major surgery. This anomaly should be taken into consideration during surgical procedures around the esophagus, such as esophagectomy [4]. The impact of double SVC on differential diagnosis, specifically as a potential cause of a widened mediastinum, highlights the significance of double SVC for physicians [6]. Also, double SVC poses challenges in the placement of a central venous catheter and a pacemaker. This finding highlights the necessity and cruciality of the recognition and management of such complexities in patients with respiratory and vascular conditions.

## Discussion

The current study focuses on the raveled nature of DVA in adults, highlighting the challenges related to the recognition and management. These anomalies become very rare and diagnostically elusive when discovered in adulthood, compared to the prenatal and early childhood days [7]. As discussed in the three cases, due to the absence of unique clinical characteristics, the rarity of DVAs in the late-onset age group often leads to incorrect diagnosis and delays the appropriate intervention [8].

Detailed imaging studies are important for an accurate diagnosis. Concurrent mitral stenosis with an absent right-side pulmonary artery underscores the importance of such a detailed imaging study. Clinicians should be aware of undiagnosed cases of isolated absence of unilateral pulmonary artery in adult patients with unexplained hemoptysis or exertional dyspnea [9]. Chest X-ray findings, including tracheal deviation, obscured hilar shadow, and loss of lung volume, played a pivotal role in providing clues for alternate diagnosis and possibilities of anomaly, which can be confirmed by contrast-enhanced computed tomography (CECT) or CT pulmonary angiogram. When COPD and the absent LPA co-exist, the requirement for a multidisciplinary strategy for the management of complex anomalies in individuals is emphasized. Treatment options range from follow-up for asymptomatic cases to surgical interventions for severe complications [10,11]. These complications include persistent hemoptysis despite repeated embolization, recurrent infections where pneumonectomy has proven successful, and pulmonary artery hypertension that is uncontrolled with medications, where the two-segment technique has shown to be beneficial [12].

The final case study included chronic respiratory conditions, such as bronchial asthma and pulmonary tuberculosis with vascular anomalies. The imaging findings of persistent SVC on the left side and an aberrant right-sided subclavian artery presenting with volume loss of the right-sided lung highlight the complexity of this case, indicating the requirement of a comprehensive evaluation and a multidisciplinary approach for accurate diagnosis and tailored management [13,14]. Among the general population, the incidence of double SVC is approximately 0.3%, but in patients with congenital heart disease, it increases significantly between 10% and 11% [15-17].

The central theme of the discourse focuses on the pivotal function of sophisticated imaging methods in elucidating the intricacies of DVAs in adults. Furthermore, multidisciplinary collaboration between clinicians and radiologists is indispensable for successful diagnosis and effective management strategies in this distinctive patient group. This series of cases adds valuable insights into the medical community. It also highlights the necessity of heightened clinical suspicion and rigorous evaluation when faced with atypical respiratory symptoms and abnormal imaging findings in adult patients, like an obscured hilar shadow with a shrunken affected lung and shift of the mediastinal structures to the affected side on chest X-rays [18].

## Conclusions

Clinicians and radiologists should be aware of the potential emergence of DVAs during adulthood. These anomalies may remain dormant until the later phases of life, emphasizing the significance of sustained vigilance in medicine. When faced with unexplained abnormalities on chest X-rays, such as loss of lung volume on one side and recurrent infections, a heightened sense of suspicion is crucial. This proactive approach enhances the probability of early detection, enabling prompt intervention and sparing patients from unnecessary invasive procedures. Consequently, this process streamlines the diagnostic process and optimizes patient care.
